# Low-frequency exercise training improves cardiovascular fitness and strength during treatment for breast cancer: a single-arm intervention study

**DOI:** 10.1038/s41598-021-01962-4

**Published:** 2021-11-23

**Authors:** Kirsten E. Bell, Amanda G. Pfeiffer, Schuyler Schmidt, Lisa Bos, Caryl Russell, Tyler Barnes, Katie M. Di Sebastiano, Egor Avrutin, Marielle Gibson, Joel A. Dubin, Marina Mourtzakis

**Affiliations:** 1 Department of Kinesiology and Health Sciences, University of Waterloo, Waterloo, ON N2L 3G1 Canada; 2 School of Public Health Sciences, University of Waterloo, Waterloo, ON N2L 3G1 Canada

**Keywords:** Obesity, Breast cancer

## Abstract

Aerobic and resistance exercise during and after cancer treatment are important for health-related outcomes, however treatment-specific barriers may inhibit adherence. We explored the effect of lower-frequency exercise training on fitness, body composition, and metabolic markers (i.e. glucose and lipids) in a group of recently diagnosed breast cancer patients. Fifty-two females ≥ 18 years with stage I–IIIB breast cancer were instructed to attend 2 cardiovascular and strength training sessions/week over 12 weeks, but program length was expanded as needed to accommodate missed sessions. Pre- and post-intervention, we measured: (1) cardiovascular fitness, (2) isometric strength, (3) body composition (dual-energy X-ray absorptiometry), and (4) fasting glucose, insulin, c-peptide, and lipids. Pre-intervention, participants were 53 ± 10 years old (mean ± SD) and overweight (BMI: 27.5 ± 5.4 kg m^−2^, 40.1 ± 6.5% body fat). Forty participants completed the program over a median 20 weeks (range: 13–32 weeks, median frequency: 1.2 sessions/week), over which predicted VO_2_peak improved by 7% (2.2[0.1–4.4] mL/kg/min) (delta[95% CI]), and strength increased by 7–9% (right arm: 2.3[0.1–4.5] N m; right leg: 7.9[2.1–13.7] N m; left leg: 7.8[1.9–13.7] N m). Body composition and metabolic markers were unchanged. An exercise frequency of 1.2 sessions/week stimulated significant improvements in fitness, and may represent a practical target for patients during active treatment.

## Introduction

With advancements in screening and treatments for breast cancer, 5-year survival rates have reached 80–90% worldwide^1^. Anti-neoplastic treatments like chemotherapy and radiation are essential towards improving survival, but they are associated with myriad negative health consequences, including unfavourable changes in physical function^2^, body composition^3^, and quality of life^4^. Glucose and lipid metabolism also become dysregulated over the course of adjuvant therapy^5^ placing survivors (defined here as individuals who have completed treatment) at an elevated risk of developing co-morbidities, such as type 2 diabetes^6^ and even cancer recurrence^7^. Given the high 5-year survival rates for breast cancer, it is important to address these negative treatment-related changes to support health and wellbeing in survivorship.

Substantial evidence supports regular exercise during treatment for breast cancer towards improving physical function, fatigue, and quality-of-life^8–10^. Exercise training also prevents deleterious changes in body composition (fat gains and lean tissue losses)^11–16^, and may help preserve metabolic health during treatment^17^. Based on this body of work, an international panel of experts has recently issued exercise guidelines for cancer patients and survivors^9^. To reduce fatigue as well as optimize physical function and quality-of-life post-diagnosis, the expert panel recommends 8–12 weeks of: (1) ≥ 30 min of moderate-intensity aerobic exercise 3 times per week, and (2) moderate-intensity resistance training (8–15 repetitions for ≥ 2 sets) 2 times per week. However, these guidelines are based on evidence from tightly controlled efficacy (vs. effectiveness) studies^18^, and the panel acknowledges that individual patient needs may necessitate fewer weekly exercise sessions. Recently diagnosed breast cancer patients, for instance, face numerous real and perceived barriers to exercise^19^ compared with individuals who have completed treatment, and may have difficulty adhering to these guidelines.

Disease- and treatment-related barriers (e.g., doctors appointments, chemotherapy, line insertions etc.) account for the majority (> 50%) of missed exercise sessions amongst patients who initiated an exercise program near a breast cancer diagnosis^19^. Perceived barriers, including the elevated stress and anxiety associated with a cancer diagnosis^20^, also inhibit motivation to initiate and adhere to regular exercise^21^. As such, a significant proportion of patients in the real-world (i.e., outside of a research setting) may struggle to exercise 3 times per week for 12 weeks during active treatment due to medical appointments or illness. Currently, the weekly exercise frequency that is most practical for newly diagnosed patients remains unknown. A flexible exercise program that accommodates barriers to exercise may be more realistic and approachable for patients undergoing treatment. It is possible that exercising semi-regularly during treatment—but below the expert panel recommendations—still stimulates modest improvements in fitness, body composition, and metabolism. However, since most studies prescribe frequency-based exercise with firm end-dates, the physiological effects of a flexible patient-oriented exercise program are unclear.

To accommodate the real- and perceived barriers that in-person exercise training may present for recently diagnosed breast cancer patients, we conducted an exploratory single-arm intervention study. Our primary objective was to evaluate the effect of 24 sessions of supervised exercise (with a targeted time for completion that was expanded as needed to accommodate missed sessions) on cardiovascular fitness and strength in a group of biological females with recently diagnosed breast cancer. Our secondary outcomes included body composition and features of glucose and lipid metabolism. Despite the low frequency of our exercise prescription, we hypothesized that improvements in all outcomes would be observed.

## Results

### Participant characteristics

**Figure 1:**
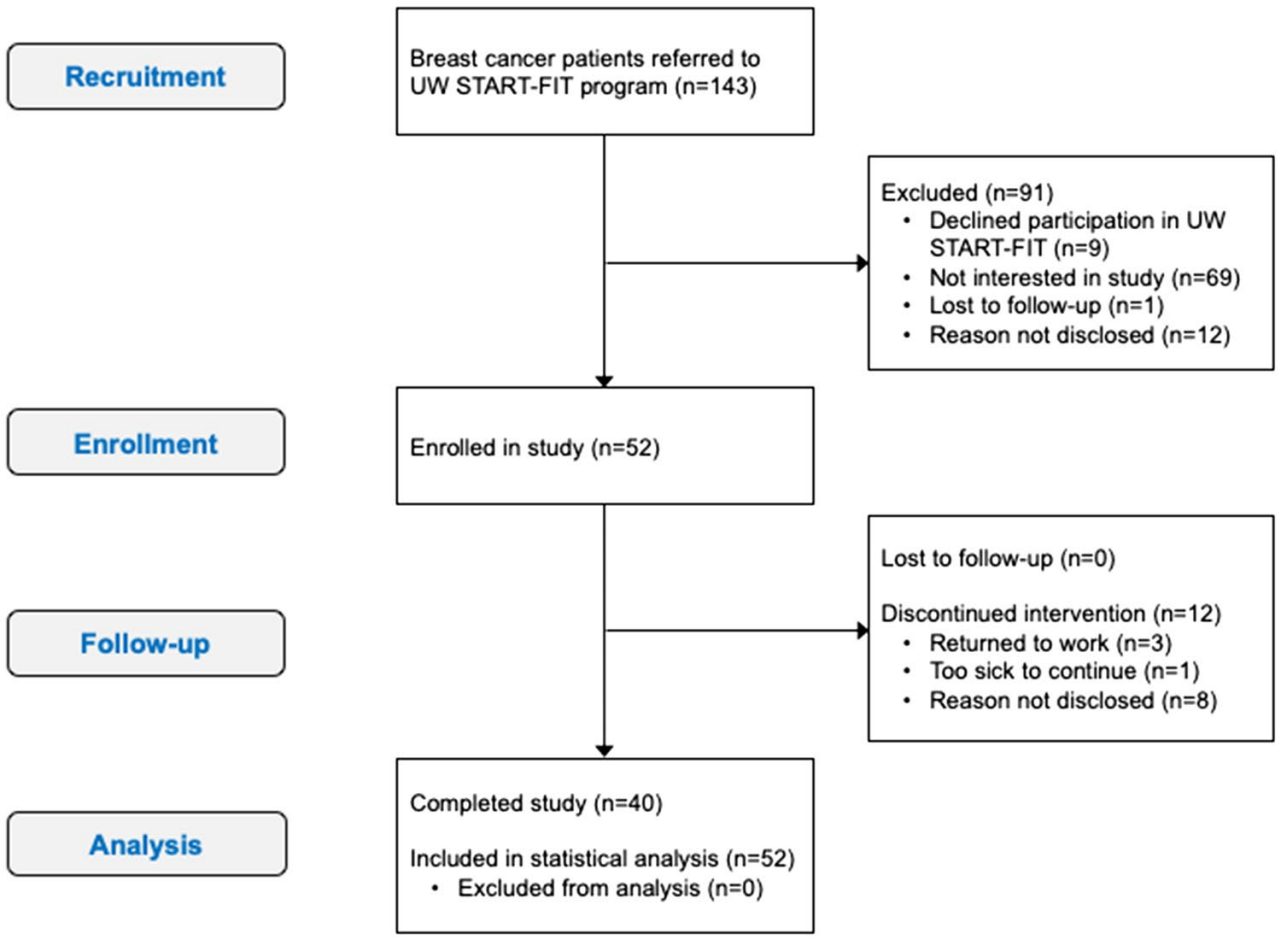
CONSORT flow diagram. Illustration of the movement of participants through the study.

Fifty-two (36%) out of 143 eligible individuals referred to UW START-FIT from our regional cancer centre consented to participate (Fig. 1). The most common reasons for not participating were declining enrolment in UW START-FIT (n = 9) and lack of interest in the study (n = 69). Post-assessments were completed on 40 (77%) of the 52 participants who began the study; the remaining 12 participants withdrew partway through the exercise program. Most individuals who withdrew did not disclose a reason for leaving the study (n = 8); the remaining non-completers returned to work (n = 3) or became too sick to continue (n = 1).

At baseline, participants were 53 ± 10 years of age, overweight based on BMI (27.5 ± 5.4 kg m^−2^), and abdominally obese (waist circumference: 94.3 ± 13.7 cm) based on criteria from the International Diabetes Federation (IDF; Table 1). Whole-body percent fat was 40.1 ± 6.5% (Table 1). As shown in Table 2, most participants presented with unilateral cancer of the left breast (left: 58%, right: 36%, bilateral: 6%). Evidence of lymphedema was observed in 2 participants. Surgery (92%) and radiation (87%) were the most common forms of treatment, followed by chemotherapy (71%) and hormonal therapy (63%). A smaller proportion of participants received targeted (i.e., monoclonal antibody) therapy (25%). Four participants were treated exclusively with surgery and/or hormonal therapy. All participants had recently initiated treatment prior to baseline assessments (e.g., surgery and/or up to 2 cycles of chemotherapy), and continued their respective treatment regimens through the post-intervention assessments.

**Table 1:** Baseline characteristics. Data are mean ± SD unless otherwise indicated.

	All participants (n = 52)	Completers (n = 40)	Non-completers (n = 12)	Healthy range^a^
**Physical and body composition characteristics**				
Age, y	53 ± 10	53 ± 10	54 ± 9	–
Weight, kg	72.5 ± 14.1	72.3 ± 15.0	73.2 ± 10.8	–
Height, m	1.62 ± 0.07	1.62 ± 0.06	1.63 ± 0.09	–
BMI, kg m^−2^	27.5 ± 5.4	27.5 ± 5.5	27.8 ± 5.2	–
< 18.5 (n, %)	0, 0%	0, 0%	0, 0%	–
18.5–24.9 (n, %)	20, 38%	16, 40%	4, 33%	18.5–24.9
25.0–29.9 (n, %)	16, 31%	11, 27.5%	5, 42%	–
30.0–34.9 (n, %)	13, 25%	11, 27.5%	2, 17%	–
≥ 35.0 (n, %)	3, 6%	2, 5%	1, 8%	–
Waist circumference, cm	94.3 ± 13.7	93.9 ± 14.2	95.5 ± 12.6	< 88
≥ 88 cm (n, %)	31, 60%	22, 56%	9, 75%	–
FM, %	40.1 ± 6.5	39.8 ± 7.0	41.3 ± 4.9	
LSTM, kg	39.6 ± 5.6	39.7 ± 6.1	39.5 ± 3.9	
**Cardiovascular fitness**				
**Resting**				
HR, bpm	72 ± 12	72 ± 13	73 ± 10	–
SBP, mmHg	118 ± 16	117 ± 15	118 ± 19	< 130
DBP, mmHg	77 ± 10	77 ± 10	75 ± 12	< 85
**GXT (final stage)**				
Workrate, W	77 ± 26	76 ± 27	80 ± 22	–
HR, bpm	127 ± 17	127 ± 17	130 ± 16	–
SBP, mmHg	164 ± 21	166 ± 21	155 ± 20	–
DBP, mmHg	76 ± 13	78 ± 11	75 ± 16	–
VO_2_peak, mL kg^−1^ min^−1^	31.9 ± 8.9	32.1 ± 9.5	31.0 ± 6.7	–
< Fair (n, %)	6, 12%	6, 15%	0, 0%	–
≥ Fair (n, %)	46, 88%	34, 85%	12, 100%	≥ Fair^b^
**Isometric strength**				
Right biceps, N m	32 ± 11	32 ± 11	33 ± 9	–
Left biceps, N m	31 ± 11	31 ± 12	30 ± 7	–
Right quadriceps, N m	101 ± 29	100 ± 29	102 ± 29	–
Left quadriceps, N m	102 ± 27	100 ± 28	105 ± 24	–
**Glucose and lipid metabolism**				
HbA1c, %	5.74 ± 0.83	5.62 ± 0.65	6.13 ± 1.24	≤ 5.6
Fasting serum glucose, mM	4.89 ± 1.17	4.87 ± 0.95	4.99 ± 1.88	< 5.6
Insulin, μIU mL^−1^	17.2 ± 10.3	17.9 ± 11.3	14.5 ± 4.2	–
HOMA-IR	4.1 ± 4.3	4.3 ± 4.7	3.3 ± 1.9	–
C-peptide, ng mL^−1^	2.37 ± 1.08	2.3 ± 1.1	2.9 ± 1.0	–
HDL-c, mM	1.32 ± 0.37	1.31 ± 0.37	1.37 ± 0.37	≥ 1.3
TC/HDL-c	3.63 ± 1.30	3.73 ± 1.21	3.30 ± 1.60	–
LDL-c, mM	2.60 ± 1.00	2.69 ± 1.01	2.29 ± 0.91	< 2.6
TAG, mM	2.90 ± 1.50	3.03 ± 1.49	2.43 ± 1.56	< 1.7

No significant differences between completers and non-completers.

^a^Based on reference values established by the International Diabetes Federation^35^ (IDF; waist circumference, HbA1c, fasting serum glucose, HDL-c, TAG, and resting SBP and DBP), the National Cholesterol Education Program Adult Treatment Panel III^36^ (NCEP ATP III; TC, LDL-c), and Baumgartner et al. (1998)^37^ (ALMI).

^b^Classification of cardiovascular fitness (very poor, poor, fair, good, excellent, superior) is based on age- and sex-specific normative values established by the American College of Sports Medicine.

*ALMI* appendicular lean mass index, *BMI* body mass index, *FM* fat mass, *DBP* diastolic blood pressure, *HbA1c* glycated hemoglobin, *HDL-c* high-density lipoprotein cholesterol, *HOMA-IR* homeostatic model assessment of insulin resistance, *HR* heart rate, *LDL-c* low-density lipoprotein cholesterol, *LSTM* lean soft tissue mass, *SBP* systolic blood pressure, *TAG* triglycerides, *VO*_*2*_*peak* peak oxygen uptake.

**Table 2:** Clinical and treatment characteristics.

	All participants (n = 52)	Completers (n = 40)	Non-completers (n = 12)
**Side affected**			
Right	19	13	6
Left	30	25	5
Bilateral	3	2	1
**Evidence of lymphedema**			
Yes	2	0	2
No	50	40	10
**Surgery**	**48, 92%**	**38, 95%**	**10, 83%**
Lumpectomy	23	18	5
Mastectomy	17	13	4
Wedge resection	7	6	1
Unspecified	1	1	0
Lymph node dissection	24	20	4
**Radiation**	**45, 87%**	**37, 93%**	**8, 67%**
**Chemotherapy**	**37, 71%**	**28, 70%**	**9, 75%**
ACT	22	19	3
CT	5	4	1
AC	1	1	0
Paclitaxel only	4	2	2
Docetaxel and carboplatin	2	1	1
FEC-D	1	1	0
Unspecified	2	0	2
**Hormonal Therapy**	**33, 63%**	**27, 68%**	**6, 50%**
Tamoxifen	12	11	1
Anastrozole	8	7	1
Letrozole	5	2	3
Exemestane	1	1	0
Unspecified	9	7	2
**Targeted therapy**	**13, 25%**	**9, 23%**	**4, 33%**
Trastuzumab	13	9	4
Pertuzumab	4	2	2

Data are n, %

No significant differences between completers (n = 40) and non-completers (n = 12).

*AC* doxorubicin (Adriamycin) and cyclophosphamide (Cytoxan), *ACT* doxorubicin (Adriamycin) and cyclophosphamide (Cytoxan), followed by paclitaxel (Taxol) or docetaxel (Taxotere), *CT* cyclophosphamide (Cytoxan) and paclitaxel (Taxol) or docetaxel (Taxotere), *FEC-D* 5-fluorouracil-epirubicin-cyclophosphamide followed by docetaxel.

Bold is used to highlight the number and proportion of patients who received each major class of treatment (e.g., sugergy, radiation, chemotherapy etc.), versus individual drugs or forms of surgery.

There were no differences in any baseline physical or clinical characteristics between participants who completed pre- and post-assessments (completers: n = 40) and those who withdrew (non-completers: n = 12).

### Exercise program attendance

Thirty-one (78%) of the 40 completers attended all 24 sessions, and all 40 completers attended at least 21 (87.5%) sessions. The 40 completers took a median of 20 weeks (range: 13–32 weeks) to complete the exercise training program. The median exercise frequency was 1.2 sessions per week (range: 0.7–1.8 sessions per week). On average, the completers attended 98.6 ± 2.9% of the prescribed 24 sessions. Disease- or treatment-related issues accounted for 76% of missed or rescheduled exercise sessions. Specific reasons included (in order from most to least common): feeling too sick to exercise (flu, nausea, faintness, joint pain), scheduled surgery, and medical appointments (chemotherapy, port removal). Non-cancer reasons accounted for 21% of missed or rescheduled sessions (e.g., vacation, inclement weather, childcare duties). No reason was disclosed for 3% of missed or rescheduled sessions. The frequency of missed or rescheduled sessions was relatively evenly distributed throughout the program: on average, sessions 1 through 24 were missed or rescheduled 8.2 ± 2.5 times. The average interval between sessions was 5.6 ± 1.2 days and did not differ throughout the program (*p* = 0.295).

The 12 non-completers withdrew from the study after a median of 6 weeks (range: 0–18 weeks). The median exercise frequency for the non-completers was 0.7 sessions per week (range: 0.0–2.0 sessions per week). On average, the non-completers attended 32.6 ± 31.2% of the prescribed 24 sessions; none completed the post-intervention assessments.

Available pre- and post-intervention data from all participants (n = 52) was included in the final statistical analysis (see “Methods” section below).

### Cardiovascular fitness and strength

Following the exercise intervention, we observed a significant 10 W increase in workrate (95% CI 5–15 W) during the final stage of the graded exercise test. We also observed a significant 4 mmHg decrease in resting DBP (95% CI − 6 to − 1 mmHg). Predicted VO_2_peak increased by 2.2 mL/kg/min (95% CI 0.1–4.3 mL/kg/min). We observed significant 7–9% increases in right biceps (2.3 N m, 95% CI 0.1–4.5 N m, Table 3), and right (7.9 N m, 95% CI 2.1–13.7 N m) and left (7.8 N m, 95% CI 1.9–13.7 N m) quadriceps isometric force production. Left biceps strength increased by 1.9 N m, but this change did not reach the threshold for statistical significance (95% CI − 0.4 to 4.2 N m).

**Table 3:** Pre- and post-intervention measures.

	Pre	Post	Mean change (95% CI)
**Physical and body composition characteristics**			
Age, y	53.0 ± 10.1	53.1 ± 10.3	0.0 (− 1.4, 1.5)
Weight, kg	72.5 ± 14.1	72.5 ± 15.7	0.1 (− 1.6, 1.8)
Height, m	1.62 ± 0.69	1.62 ± 0.06	0.19 (− 1.12, 1.50)
BMI, kg m^−2^	27.5 ± 5.4	27.4 ± 5.8	− 0.2 (− 1.1, 0.6)
Waist circumference, cm	94.3 ± 13.7	93.8 ± 15.1	− 0.5 (− 3.0, 2.0)
FM, %	40.1 ± 6.5	44.9 ± 6.9	0.1 (− 1.0, 1.1)
LSTM, kg	39.6 ± 5.6	39.5 ± 6.1	0.2 (− 1.0, 1.3)
**Cardiovascular fitness**			
**Resting**			
HR, bpm	72 ± 12	70 ± 12	− 2 (− 5, 1)
SBP, mmHg	118 ± 16	114 ± 15	− 4 (− 8, 1)
DBP, mmHg	77 ± 10	74 ± 9	− **4 (**− **6,** − **1)**
**GXT (final stage)**			
Workrate, W	77 ± 26	87 ± 32	**10 (5, 15)**
HR, bpm	127 ± 17	128 ± 19	0 (− 4, 4)
SBP, mmHg	164 ± 21	160 ± 20	− 3 (− 9, 2)
DBP, mmHg	76 ± 13	77 ± 9	0 (− 3, 3)
Predicted VO_2_peak, mL kg^−1^ min^−1^	31.9 ± 8.9	34.0 ± 9.4	**2.2 (0.1, 4.3)**
**Isometric strength**			
Right biceps, N m	32 ± 11	34 ± 11	**2.3 (0.1, 4.5)**
Left biceps, N m	31 ± 11	33 ± 11	1.9 (− 0.4, 4.2)
Right quadriceps, N m	100 ± 29	109 ± 30	**7.9 (2.1, 13.7)**
Left quadriceps, N m	102 ± 27	110 ± 31	**7.8 (1.9, 13.7)**
**Glucose and lipid metabolism**			
Fasting serum glucose, mM	4.89 ± 1.17	4.62 ± 0.52	− 0.23 (− 0.56, 0.09)
Insulin, μIU mL^−1^	17.2 ± 10.3	18.0 ± 13.53	1.1 (− 0.4, 2.6)
HOMA-IR	4.1 ± 4.3	3.9 ± 3.4	− 0.2 (− 1.0, 0.6)
C-peptide, ng mL^−1^	2.37 ± 1.08	2.18 ± 1.08	− 0.18 (− 0.51, 0.16)
HDL-c, mM	1.32 ± 0.37	1.43 ± 0.41	0.14 (− 0.07, 0.35)
TC/HDL-c	3.63 ± 1.30	3.63 ± 1.45	0.04 (− 0.37, 0.46)
LDL-c, mM	2.60 ± 1.00	2.77 ± 1.1	0.11 (− 0.18, 0.40)
TAG, mM	2.90 ± 1.50	3.01 ± 1.51	0.15 (− 0.56, 0.85)

Data are mean ± SD unless otherwise indicated.

Pre- and post-intervention measurements were compared using 2-tailed paired t-tests on pooled multiple imputation data.

Bolded values indicate statistically significant changes.

*ALMI* appendicular lean mass index, *BMI* body mass index, *DBP* diastolic blood pressure, *FM* fat mass, *FFM* fat-free mass, *HDL-c* high-density lipoprotein cholesterol, *HOMA-IR* homeostatic model assessment of insulin resistance, *HR* heart rate, *LDL-c* low-density lipoprotein cholesterol, *SBP* systolic blood pressure, *TAG* triglycerides; *VO*_*2*_ oxygen uptake.

### Body composition and metabolism

We did not observe any changes in anthropometric (weight, BMI, waist circumference) or DXA-based measurements (FM, LSTM) of whole-body or regional body composition over the course of exercise training (see Table 3).

Prior to the exercise intervention, mean HbA1c (5.74 ± 0.83%, healthy range: ≤ 5.6%) and fasting TAG (2.90 ± 1.50 mM, healthy range: < 1.7 mM), and LDL-c (2.60 ± 1.00 mM, healthy range: < 2.6 mM) concentrations were outside the normal range. Average serum glucose was within the healthy range at baseline (4.89 ± 1.17 mM, healthy range: < 5.6 mM). Following exercise training, we did not detect any statistically significant changes in fasting markers of glucose or lipid metabolism (Table 3). Serum glucose decreased by 5% (0.23 mM, 95% CI − 0.56 to 0.09 mM) and HDL-c increased by 10% (0.14 mM, 95% CI − 0.07 to 0.35 mM), but these changes did not achieve statistical significance.

## Discussion

In this proof-of-concept single-arm study, we examined the effectiveness of a flexible exercise program, consisting of 24 sessions of combined exercise training with a targeted frequency of 2 sessions per week, on cardiovascular fitness and strength in a group of biological females with recently diagnosed breast cancer. We expanded the length of the program as needed to accommodate participants’ medical appointments (for surgery, chemotherapy, port removal) and treatment-related side-effects, such as nausea and faintness. Participants completed the exercise intervention over a median of 20 weeks, and attended training sessions at a median frequency of 1.2 sessions per week. Although this frequency was lower than targeted, our study demonstrated that exercising < 2 times per week was sufficient to stimulate significant cardiovascular fitness and strength adaptations. Further, our intervention may have prevented the anticipated development of deleterious treatment-related changes to body composition and metabolism. These findings form the foundation for future, larger-scale pragmatic studies in which we will further explore this hypothesis.

Cardiovascular fitness and strength improved despite the low frequency of exercise training sessions. Although we prescribed a total of 24 exercise sessions at a targeted frequency of 2 sessions per week, participants only attended a median of 1.2 exercise sessions per week over 20 weeks. No participants completed the study within in the targeted time frame of 12 weeks. Importantly, however, the frequency of missed or rescheduled sessions was distributed relatively evenly throughout the program, suggesting that participants exercised regularly and did not miss numerous consecutive sessions. The median frequency of 1.2 sessions per week was lower than current expert panel recommendations for individuals undergoing active treatment for cancer (aerobic exercise 3 times per week plus resistance exercise 2 times per week)^9^. Despite that our participants did not meet these recommendations, our intervention may have been sufficient to stimulate significant improvements in cardiovascular fitness, blood pressure, and strength. These improvements are particularly important, considering that VO_2_peak and grip strength are inversely correlated with cancer-specific and all-cause mortality^22,23^. Future work should investigate whether behavioural supports (e.g., physical activity and/or diet counselling) and home-based exercise options may improve adherence to recommendations.

Previous work has shown that predicted VO_2_peak decreases by 10–33% in breast cancer patients during 12–16 weeks of chemotherapy^24,25^. Chemotherapy is also associated with decrements of 5–9% in upper and lower body isometric force production^24^. Our exercise intervention not only prevented these potential decreases in physical fitness but resulted in significant improvements in resting blood pressure, final-stage workrate during a graded exercise test, and predicted VO_2_peak. We also observed significant increases in right biceps and left and right quadriceps isometric strength. Left biceps strength did not increase, however, this is likely because 33/52 (63%) participants had cancer in their left breast and may have been unable or hesitant to lift heavier loads post-surgery or during ongoing localized radiation treatment. Future studies that include a usual care group are needed to confirm our findings. Additionally, we recognize that since most (71%), but not all, patients in this study received chemotherapy, our cohort may have been less vulnerable to the aforementioned decrements in VO_2_peak and strength associated with certain chemotherapeutic agents^24,25^. Given the heterogeneity of treatment regimens in the present study, we performed a sensitivity analysis to confirm that improvements were not driven by participants who received less intensive treatment. This posthoc analysis revealed that censoring the 4 participants who were treated only with surgery and/or hormonal therapy (which are generally less fatigue-inducing that chemotherapy or radiation) did not alter the main findings of our study. We are therefore confident that these improvements in fitness are attributable to our exercise intervention, especially since participants were instructed not to participate in any other structured exercise program for the duration of this study. Considering that certain breast cancer patients have been shown to lose strength during treatment, the maintenance of left arm strength and improvements in cardiovascular fitness and blood pressure outcomes indicate a beneficial physiological response to exercise training in individuals undergoing treatment for breast cancer.

### Body composition and markers of glucose and lipid regulation were maintained during the study

Fasting concentrations of glucose, insulin, cholesterols, and triglycerides were maintained over the course of exercise training. Numerous studies have reported significant deleterious perturbations to glucose^5,26,27^ and lipid^5,28,29^ metabolism during adjuvant treatment for breast cancer. Specifically, fasting serum glucose^5^ and insulin^5,26,27^ concentrations have been shown to increase by ~ 20% and ~ 20–75%, respectively, within the first 6 months following diagnosis. Unhealthy lipid partitioning^5,26,28^ (increased LDL-c and TAG concentrations, and/or decreased HDL-c concentrations) has also been observed early in the treatment trajectory. Consistent with these findings, Dieli-Conwright et al.^5^ observed that 72.5% of newly diagnosed participants developed metabolic syndrome (a cluster of characteristics that increases the risk for type 2 diabetes and cardiovascular disease) after 12–18 weeks of adjuvant chemotherapy. The authors also observed significant negative treatment-related changes in all 5 components of metabolic syndrome (waist circumference, resting blood pressure, and fasting blood glucose, TAG, and HDL-c concentrations). Conversely, in the present study, we observed that metabolic outcomes did not worsen during treatment, likely a result of our exercise intervention.

Although metabolic health and body composition were maintained in our cohort of participants, several potential mechanisms may have prevented our capacity to detect exercise-mediated *improvements* in these outcomes. Participants exercised at a frequency lower than what has previously been demonstrated to elicit losses in fat mass during treatment (1.2 vs. 3 sessions per week)^12^. But, given that the most common reasons for missed or rescheduled sessions were treatment-related illness and medical appointments, the exercise frequency observed in our intervention may be more realistic of patients early in the treatment trajectory. Individuals with newly diagnosed breast cancer also experience elevated stress and anxiety^20^, which can further inhibit motivation to initiate and adhere to an exercise program^21^. As such, our findings are important towards understanding how flexible exercise programs may impact outcomes of interest, like fitness, body composition, and metabolism, outside of controlled research studies (i.e., in the “real world”). Despite that participants exercised at a lower frequency (median of 1.2 sessions per week), glucose and lipid outcomes did not worsen. Perhaps the addition of a concurrent nutrition intervention to manage energy, fat, and protein intake would have supported improvements in markers of glucose and lipid metabolism. In future, a larger-scale study that includes a usual care group would confirm that these changes are specifically attributable to the exercise intervention. Our smaller-scale findings are promising: if harmful metabolic and body composition changes can be prevented during treatment, rather than corrected later in treatment or in survivorship, breast cancer patients who enter survivorship may be at a reduced risk of developing secondary conditions, such as type 2 diabetes, cardiovascular disease, or even cancer recurrence.

There are several limitations to the present study. Firstly, we recruited a relatively heterogeneous group of participants: any female ≥ 18 years with stage I-IIIB breast cancer, regardless of menopausal status or treatment type, was eligible to participate. Second, our lack of a usual care reference group may have limited our ability to detect true longitudinal changes in our intervention group (and between-group differences in body composition and metabolism). Furthermore, our small sample size may have increased the susceptibility of our study to type I errors. However, this study was hypothesis-generating and builds the foundation for our future work, which will test the effect of a practical exercise program on fitness, body composition, and metabolic health during treatment for breast cancer using a randomized controlled design.

In summary, we observed that 1.2 sessions per week of combined aerobic and strength training significantly improved cardiovascular fitness and strength in a group of females with newly diagnosed breast cancer. Further, body composition and features of glucose and lipid metabolism did not worsen over the course of treatment, possibly as a result of exercise training. This is the first study to allow participants unlimited time to complete a fixed number of sessions, as a pragmatic means of accommodating cancer-specific barriers to exercise. This approach may improve the generalizability of our findings. Future larger-scale studies that include a usual care group are needed to confirm this preliminary study.

## Methods

### Screening and recruitment

Fifty-two biological females undergoing any treatment for breast cancer participated in this single-arm exercise intervention study. Any female ≥ 18 years with recently diagnosed breast cancer (stages I–IIIB, any type) was eligible to participate. Oncologists consecutively referred patients to the University of Waterloo (UW) START-FIT program unless the patient was cautioned to avoid exercise. This study received clearance from the University of Waterloo Office of Research Ethics Committee and the Tri-Hospital Research Ethics Board, and was performed in accordance with the 1964 Declaration of Helsinki and all its later amendments. All participants were informed of the nature and possible risks of the experimental procedures before their written informed consent was obtained. Full details concerning the flow of participants through this study can be found in Fig. 1.

### Experimental design

Eligible participants were enrolled in a 24-session exercise training program. The following assessments were conducted at baseline and post-intervention: cardiovascular fitness, isometric strength, and body composition. We also obtained a fasting blood sample to measure features of glucose and lipid metabolism. Due to scheduling and the availability of personnel, most post-assessments took place 5 days after the cessation of exercise training. Recent work suggests that significant detraining effects are not detectable until 1–2 weeks post-intervention^30^.

### Cardiovascular fitness assessments

Participants performed a graded exercise test on a cycle ergometer (Ergoselect 100, Ergoline; Blitz, Germany). Heart rate (HR) was measured continuously using a 5-lead electrocardiogram, and manual blood pressure and rating of perceived exertion (RPE) were assessed every two minutes. Following a 2-min warm-up at 25 W, the load was increased by approximately 20 W every two minutes. Participants were instructed to maintain a cadence of > 50 rpm. Tests were terminated if: (1) the cadence dropped below 50 rpm for > 30 s, (2) participants reported an RPE ≥ 15, (3) participants achieved > 85% of their age-predicted HRmax, or (4) volitional fatigue was attained. Maximal workrate was extrapolated using each participant’s age-predicated maximum HR and a plot of at least 2 submaximal workrates versus corresponding measured HR. VO_2_peak was estimated using the extrapolated maximal workrate and the ACSM’s prediction equation for leg ergometry^31^.

### Strength assessments

Unilateral isometric strength of the biceps (forearm flexion) and quadriceps (knee extension) was measured using a linear variable differential transformer (LVDT) attached to a cable. Participants were secured in a seated position for all measurements. Forearm flexion and knee extension were assessed with the elbows and knees, respectively, fixed at 90° flexion. Participants were verbally encouraged to contract maximally for 2 s. Peak torque was measured 3 times in each limb, with a 2-min break between each trial. The highest value was recorded and used for analysis.

### Exercise training

The UW START-FIT program is a twice weekly supervised and progressive exercise training program within our larger UW WELL-FIT Cancer Exercise Portfolio. UW START-FIT is offered exclusively to individuals during treatment for cancer who live in the Waterloo region. We instructed our study participants to attend 24 sessions, with a target frequency of twice per week for 12 weeks. Importantly, there was no maximum time limit to the program: missed sessions due to medical appointments, surgery, illness (etc.) could be made up to help participants complete all 24 training sessions in the shortest possible time. Each exercise session included both cardiovascular and whole-body strength training, and was prescribed by a Certified Exercise Physiologist based on each participant’s baseline fitness assessments. Exercise training sessions lasted approximately 1 h each. Participants wore HR monitors (Polar; Lachine, QC) for the full duration of each exercise session to ensure they remained within their target HR of 40–60% HR reserve (HRR, approximately 11–13 RPE). The number of sessions completed as well as total time elapsed between the first and last sessions were recorded.

Resting HR and blood pressure were measured prior to each exercise session to ensure participants’ safety. If resting HR exceeded 100 bpm the exercise prescription was revised, and if a participant also reported shortness of breath or unusual fatigue the exercise session was postponed. If resting blood pressure exceeded 160/94 bpm the participant was referred to a physician for medical clearance.

After a 3–5 min warm-up at a low-intensity, participants performed 15–30 min of moderate-intensity (40–60% HRR) cardiovascular exercise on a treadmill (walking), recumbent bike, or elliptical. Speed, incline and/or load (depending on equipment used) were adjusted as needed to ensure participants remained within their target HR. Cardiovascular exercise was always concluded with a 3–5 min cool-down at a low-intensity. Each participant received an individualized strength training prescription that targeted all major muscle groups (quadriceps, hamstrings, gluteals, chest, back, shoulders, arms and core). For variety and to ensure adequate training stimulus, participants received a new set of strength training exercises after the first 12 sessions were completed. Participants performed 1–2 sets of 10–20 repetitions of each exercise. The load was increased when participants could easily complete 20 repetitions of a given exercise with proper form on 2 consecutive sessions.

### Body composition

Waist circumference was measured at the top of the iliac crests using a tape measure. Whole-body and regional fat mass (FM) and lean soft tissue mass (LSTM; fat- and bone-free mass) were measured using dual-energy X-ray absorptiometry (DXA) (Hologic Discovery QDR 4500; Christie Group, Mississauga ON). Prior to all scans, a certified medical radiation technologist (MRT) performed quality control and phantom calibration procedures using phantoms provided by the manufacturer. Participants then changed into a cloth hospital gown, and weight and height were measured using a balance-beam scale and stadiometer, respectively. Participants were positioned supine on the scanning table with their shoulders depressed and forearms positioned parallel to the bed. Participants’ legs were extended with their toes internally rotated and held in position with masking tape to prevent movement during the scan. A second scan was required for participants (n = 2) who did not fit within the lateral limits of the scanning table. These scans were analyzed by summing the left limbs, trunk, and head of one scan, and the right limbs of the second scan, as previously described^32^.

### Blood sampling and biochemical analysis

The morning after an 8–12 h overnight fast, approximately 40 mL of venous blood was drawn into evacuated collection tubes. For baseline assessments only, fasting HbA1c (Roxon; Etobicoke, ON) was measured in fresh whole blood immediately upon collection. The blood was then allowed to clot at room temperature for a minimum of 30 min before centrifugation. The serum was aliquoted and frozen at − 80 °C for batch analysis.

We measured glucose, insulin, c-peptide and lipid panel (high-density lipoprotein cholesterol [HDL-c], low-density lipoprotein cholesterol [LDL-c], and triglycerides [TAG]) in serum collected at baseline and post-intervention. Glucose was measured spectrophotometrically using the glucose oxidase method. HDL-c and TAG were measured with commercially available kits (BioPacific Diagnostic Inc; Vancouver, BC), and LDL-c was calculated based on the total, HDL-c, and TAG content of each sample. Insulin and c-peptide were measured using radioimmunoassay kits (Millipore Sigma; Oakville, ON). All samples were analyzed in a minimum of 2 replicates, and samples with coefficients of variation (CV) > 5% (for glucose and all lipids) or > 10% (for insulin and c-peptide) were rerun. The average CVs for these assays were 2.2 ± 2.2% for glucose, for 5.1 ± 3.5% insulin, 3.8 ± 2.5% for c-peptide, 1.1 ± 2.2% for total cholesterol, 3.0 ± 2.5% for HDL-c, and 2.3 ± 2.4% for TAG.

### Statistical analysis

Statistical analysis was completed using SPSS (IBM SPSS Statistics for Mac, version 26.0; IMB Corp.; Armonk, NY). We compared baseline characteristics between completers (participants who completed pre- and post-intervention assessments, n = 40) and non-completers (participants who withdrew from the study, n = 12) using two-tailed independent samples t-tests for continuous variables and *Χ*^2^ tests for categorical variables.

Overall, 17.8% of all data was missing. The percentage of missing data ranged from 0% for some anthropometric variables to 40% for certain blood markers (for details please refer to supplementary Table S1). The most common reasons for missing data at baseline were technical difficulties obtaining blood samples and scheduling conflicts. Withdrawal from the study accounted for 58–100% of missing data post-intervention. All data were missing completely at random (MCAR), according to Little’s MCAR test. Therefore, based on recommendations for longitudinal human studies^33^, we addressed the problem of missing data using the multiple imputation function (automatic method) in SPSS. Pre- and post-intervention outcomes were then compared using two-tailed paired t-tests on data pooled from five imputations in accordance with Rubin’s rules^34^. Imputed values were similar to observed values, so observed values are presented in the Results section.

For all statistical analyses, significance is highlighted if *p* < 0.05. Data are presented as means ± standard deviations (SD), unless otherwise indicated.

## Supplementary Information

Supplementary Information.

## Data Availability

The datasets generated during and/or analyzed during the current study are available from the corresponding author on reasonable request.
