# Supplementary material for: Low-frequency exercise training improves cardiovascular fitness and strength during treatment for breast cancer: a single-arm intervention study
Source: Sci Rep. 2021 Nov 23;11:22758. doi: 10.1038/s41598-021-01962-4 (PMC8610997; doi:10.1038/s41598-021-01962-4)
Supplement: Supplementary file 1 — Supplementary Information. [file 41598_2021_1962_MOESM1_ESM.pdf]

Low-frequency exercise training improves cardiovascular fitness and strength during treatment for breast cancer: A single-arm intervention study

Scientific Reports

Kirsten E. Bell, Amanda G. Pfeiffer, Schuyler Schmidt, Lisa Bos, Caryl Russell, Tyler Barnes, Katie M. Di Sebastiano, Egor Avrutin, Marielle Gibson, Joel A. Dubin & Marina Mourtzakis*

*Corresponding author affiliation: Department of Kinesiology, University of Waterloo, Waterloo ON, N2L 3G1; Email: mmourtzakis@uwaterloo.ca

Table S1. Missingness for outcomes at baseline and post-intervention (n=52). Data are presented as $n_{missing}$, %.

	Baseline	Post-intervention
Physical and body composition characteristics		
Age	0, 0%	12, 23%
Weight	0, 0%	12, 23%
Height	0, 0%	12, 23%
BMI	0, 0%	12, 23%
Waist circumference	1, 2%	15, 29%
FM	2, 4%	13, 25%
LSTM	2, 4%	13, 25%
ALMI	2, 4%	13, 25%
Glucose and lipid metabolism		
HbA1c	8, 15%	n/a
Fasting serum glucose	8, 15%	20, 39%
Insulin	8, 15%	20, 39%
HOMA-IR	8, 15%	21, 40%
C-peptide	8, 15%	19, 37%
HDL-c	5, 9%	19, 37%
TC/HDL-c	5, 9%	19, 37%
LDL-c	7, 13%	29, 39%
TAG	7, 13%	19, 37%
Cardiovascular fitness		
Resting HR	2, 4%	14, 27%
Resting BP	0, 0%	14, 27%

Predicted VO_2peak	0, 0%	14, 27%
Final stage workrate	0, 0%	15, 29%
Final stage HR	0, 0%	15, 29%
Final stage BP	0, 0%	15, 29%
	Isometric strength	
Right biceps	1, 2%	14, 27%
Left biceps	2, 4%	15, 29%
Right quadriceps	1, 2%	14, 27%
Left quadriceps	1, 2%	14, 27%

Abbreviations: ALMI, appendicular lean mass index; BMI, body mass index; DBP, diastolic blood pressure; FM, fat mass; FFM, fat-free mass; HbA1c, glycated hemoglobin; HDL-c, high-density lipoprotein cholesterol; HOMA-IR, homeostatic model assessment of insulin resistance; HR, heart rate; LDL-c, low-density lipoprotein cholesterol; SBP, systolic blood pressure; TAG, triacylglycerides; VO2, oxygen uptake.
